# Plasmonic Enhanced SERS in Ag/TiO_2_ Nanostructured Film: An Experimental and Theoretical Study

**DOI:** 10.3390/mi13101595

**Published:** 2022-09-25

**Authors:** Chawki Awada

**Affiliations:** Department of Physics, College of Science, King Faisal University, P.O. Box 400, Al-Ahsa 31982, Saudi Arabia; cawada@kfu.edu.sa

**Keywords:** plasmonics, SERS, Ag/TiO_2_, MB, finite element simulation, EM enhancement

## Abstract

In this work, we present a new study on the electromagnetic (EM) enhancement properties generated by Ag/TiO_2_ toward the finger print of methylene blue (MB) molecules deposited on the surface of Ag nanostructures. SERS intensity generated by MB molecules reflects the interaction between the local electric field and their bonds. A power-dependent SERS study in order to reveal the magnitude effect of a local electric field on the vibration behavior of molecular bonds of MB was performed. A theoretical study using finite element (COMSOL Multiphysics) was performed in order to understand the effect of interparticle distance of Ag nanoparticles on the enhancement properties.

## 1. Introduction

Due to its important optical property called surface plasmon resonance, noble metals have been intensively studied in the last decades with different tools of characterizations [1,2,3,4,5]. The latter has helped different communities to use the noble metal in different applications such as biosensing, catalysis, pollutant degradation, solar cell, and hydrogen production [6,7,8,9,10,11]. However, the metal–semiconductor hybrid system has recently captured more attention due to different properties related to its thermal stability, biocompatibility, photo carrier generation, synergetic effect, etc. In addition, it is much less expensive [12,13,14,15,16,17]. 

When coupling semiconductors with metal, the plasmonic properties change drastically, e.g., the efficiency of plasmonic NPs is improved by tuning the plasmon resonance higher/lower wavelength, therefore expanding the absorption response frequency of NPs [5,18]. Localized surface plasmon resonance (LSPR) wavelength depends strongly on the gap distance between metallic nanoparticles deposited on the semiconductors. LSPR spectral range can shift and change band width by varying the gap distance between nanoparticles [19]. This is why understanding the plasmonic properties of the metal–semiconductor hybrid system is of great of interest for different applications. One important consequence of mixing two systems is using them in different applications simultaneously. For example, hybrid systems can be used in monitoring the photocatalytic activities toward sensing [20,21]. In various studies, Ag/TiO_2_ was used in different applications such as sensing via surface enhanced spectroscopy (SERS) and photocatalysis by visible light activation. 

Different studies have recently used SERS as a tool to reveal the origin of the chemical enhancement (CE) and electromagnetic (EM) enhancement in a metal–semiconductor hybrid. In fact, most of the reported studies were more focused on CE than EM enhancement. This is the reason why a new technique is needed based on SERS to study the properties of EM enhancement such as the synergistic effect and the extent of spatial distribution for localized surface plasmon. These properties can be probed via SERS signal where the vibrational mode of the probed molecules on the surface gives important insight into the EM properties, in particular, when the molecules such a MB are more energetically adsorbed into metal such as silver than TiO_2_ [22,23,24,25]. Thus, the novelty of our work consists of revealing new information on the EM enhancement using SERS. We attribute the origin of SERS to two effects: 1-the EM field generated from the surface plasmon of pure silver NPs; 2-the EM enhancement amplified and induced by the charge transfer (CT) between the silver and the TiO_2_ and vice-versa.

In this work, Ag/TiO_2_ nanostructures were fabricated by a simple chemical route. Structural analysis was carried out on the obtained samples using X-ray diffraction (XRD), field emission scanning electron microscopy (FE-SEM), and energy dispersion X-ray (EDX) mapping. SERS spectra were measured from our fabricated substrate Ag/TiO_2_ on different positions of the films. SERS spectra were studied by studying the FWHM, the shift, and the amplitude of some vibrational modes of MB. In addition, a power-dependent study was performed. Finally, we performed a simulation using finite element method to study the gap-size effect on the EM properties. 

## 2. Experimental Details

To prepare the Ag/TiO_2_, a titanium (Ti) foil (TI000410, Goodfellow, Huntingdon, England) with 250 μm thickness and 99.6% purity was used. The foil was cut into small pieces that were 2.25 cm^2^ in area, and ultrasonically well cleaned. The solution of silver nitrate (AgNO_3_) was prepared with a concentration of 40 mM. A 1.0 mL AgNO_3_ solution was wisely dropped onto the surface of the Ti foil. The sample was then placed in an alumina boat, which was placed in a furnace in ambient air. The furnace was heated from room temperature up to 800 °C in 120 min and kept at this temperature for 4.0 h. Afterward, the furnace was left to cool to the room temperature, after which the sample was collected. 

The Ag/TiO_2_ surface morphology was characterized by field emission scanning electron microscopy (FE-SEM), Model Zeiss Sigma 500 VP analytical FE-SEM (Jena, Germany) operating at 15 kV with a resolution of 0.8 nm. The same system was used for EDX mapping. The crystal structure of the prepared samples was investigated by an X-ray diffractometer (Philips Type PW 1710, Amsterdam, The Netherlands) with CuKα radiation. XRD patterns were recorded in a range of 10–80° with a scanning rate of 2°/min. Raman spectroscopy was conducted using a confocal Raman microscope (LabRAM HR800, Horiba Scientific, Villeneuve-d’Ascq, France) connected to a multichannel charge-coupled detector (CCD). A He–Ne Laser with a wavelength of 632.8 nm and 2 mW output power was used as a source of excitation. The sample was irradiated with an objective 50× We measured the Raman spectra at ambient temperature in a backscattering configuration with a spectral resolution of 0.35 cm^−1^.

## 3. Results and Discussions

### 3.1. Structural Analysis

**Figure 1:**
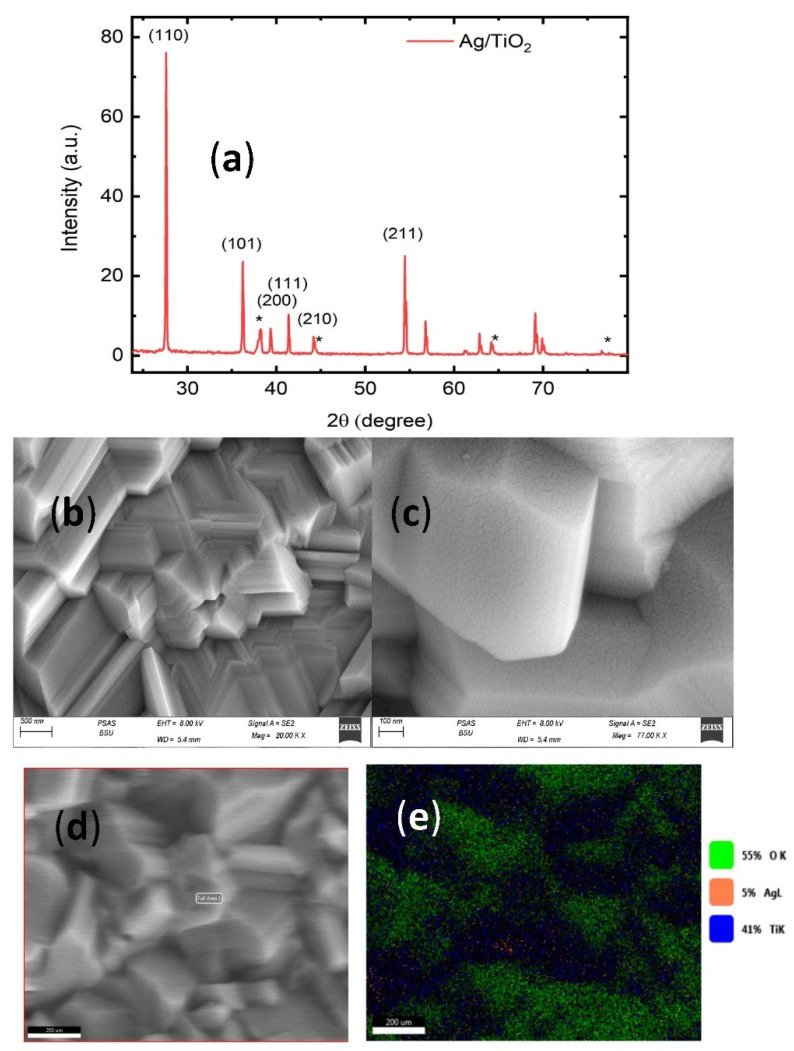
(**a**) XRD of Ag/TiO_2_ nanocrystals, * represents the XRD peaks of silver. (**b**,**c**) Top-view SEM of Ag/TiO_2_ nanocrystals with two different magnifications 20,000× and 77,000×. (**d**,**e**) EDX mapping of Ag/TiO_2_ (55% O, 5% Ag, and 41% Ti).

XRD patterns were studied to show the structure and phase composition of Ag/TiO_2_ nanocrystals. Figure 1a displays the results of XRD of Ag/TiO_2_ nanocrystals synthesized by the above described method. From the XRD pattern, it is clearly seen that the diffraction peaks at 2θ angles 27.6, 36.22, 39.34, 41.38, 44.19, and 54.44 corresponding to the crystal planes (110), (101), (200), (111), (210), and (211) belong to the fundamental rutile structure of TiO_2_ (JCPDS card no. 21-1276). In addition, some diffraction peaks at 2θ angles 38.21, 44.35, 64.7, and 77.7 are also observed in the sample, belonging to the crystal planes of silver (111), (200), (220), and (311), respectively, (JCPDS card no. 04-0783).

Field emission scanning electron microscopy (FESEM) measurement was performed on Ag/TiO_2_ nanostructures. Figure 1a,b shows the topography from the Ag/TiO_2_ surface. Figure 1b shows the appearance of formed grains with large size with multiple facets; Figure 1c shows how the silver nanoparticles are covering the surface of TiO_2_. A fractal structure is observed with space between nano islands or nanoparticles. In order to confirm the homogeneous distribution of silver into the surface of the sample, an elemental mapping of the surface of Ag/TiO_2_ surface was performed confirming the presence of 5% Ag, 55% O, and 41% Ti (see Figure 1d,e).

### 3.2. SERS on Ag/TiO_2_

**Figure 2:**
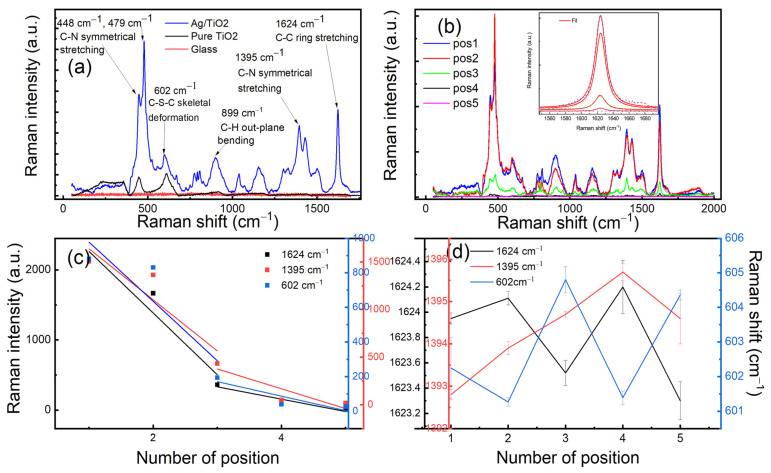
(**a**) SERS of MB measured from Ag/TiO_2_, pure TiO_2_, and glass substrate. (**b**) SERS of MB on Ag/TiO_2_ obtained from 5 points; inset shows the increasing of the intensity for 1623 cm^−1^ peak and the adjustment with a Lorentzian function. (**c**) Maximum intensities obtained at 1624 cm^−1^, 1395 cm^−1^_,_ and 602 cm^−1^ peaks at different positions. Red, blue, and black lines represent the fit of the data with a linear function. (**d**) Variation in Raman shift of the three bands 1624 cm^−1^, 1395 cm^−1^_,_ and 602 cm^−1^ versus the position’s number.

Figure 2a shows SERS spectra on the different samples: Ag/TiO_2_, pure TiO_2_, and glass. First, we tested MB on glass substrate to exclude the far-field response as an origin of the SERS signal. We can clearly see that there is no SERS signal observed into the glass. In order to assure that the silver nanoparticles are of the origin of SERS generated by MB, we performed different scans into the surface of pure TiO_2_ and Ag/TiO_2_. Consequently, we observed the effect of silver nanoparticles on the enhancement of Raman spectra of MB; however, for the pure TiO_2_, we just observed the phonon modes of the rutile phase located at 447 cm^−1^ and 610 cm^−1^. SERS spectra of MB into Ag/TiO_2_ show the appearance of the vibrational modes of MB located at 448 cm^−1^ (C-N symmetrical stretching), 479 cm^−1^ (C-N symmetrical stretching), 602 cm^−1^ (C-S-C skeletal deformation), 899 cm^−1^ (C-H out plane bending), 1395 cm^−1^ (C-N symmetrical stretching), and 1624 cm^−1^ (C-C ring stretching) [26,27]. Figure 2b shows the SERS spectra of MB on different positions of Ag/TiO_2_. It is observed the intensity changes between the position, this is related to the inhomogeneity distribution of hot spots (HS) intensity into the surface. In order to study the effect of the enhancement on the Raman lines of MB, we extracted the Raman shift and Raman intensity of different bands such as 602 cm^−1^, 1395 cm^−1^, and 1624 cm^−1^. Figure 2c shows that the intensity of the three bands decrease linearly with the same slopes for the different positions. Two regions are observed with two different slopes, the first one is between pos1 and pos3 and the second one between pos3 and pos5. In order to check the reproducibility of the spatial enhancement, we carried out two mappings (10 μm × 10 μm) of the SERS intensity for the two bands 480 cm^−1^ and 1623 cm^−1^. The two mappings are the plot of an integrated intensity under each peak (see Figure S1). 

**Figure 3:**
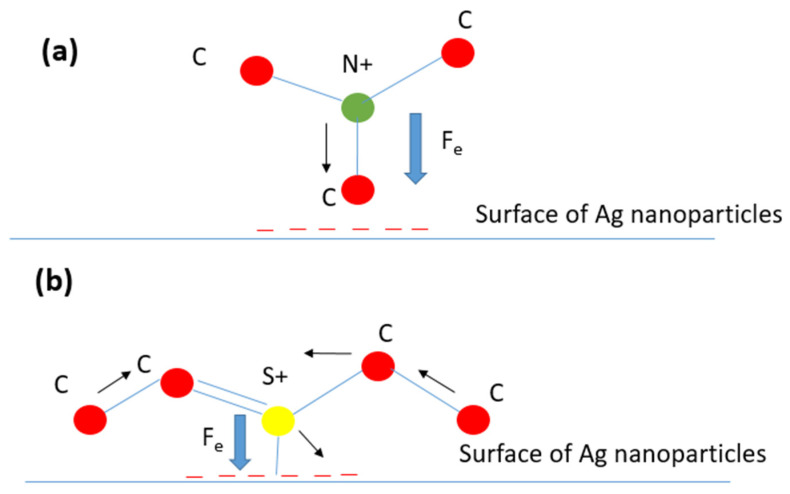
Schematic representation of the vibrational modes of MB on Ag surface. (**a**) Stretching mode of CNC bonds. (**b**) Skeletal deformation mode and stretching modes of CSC and C-C. red, green, and yellow spheres’ color represent carbon, nitrogen, and sulfur atoms, respectively.

The Raman shift variation has different features than those of the intensities. The three bands in Figure 2d present the different features. The C-N symmetrical stretching mode shifts linearly toward the blue by decreasing the intensity; this indicates a compressive stress the molecules undergo when excited by the local electrical field. The latter can be explained by two springs placed at the terminus of the MB molecule where an electrical force exists between the cations (terminus of the molecule) and the silver nanoparticles that play the role of anions (see Figure 3a). When the local electric field decreases, the electric force decreases and hence the spring retracts. The latter could explain the observed blue shift. For C-S-C skeletal deformation and C-C ring stretching modes, they are varied opposite to each to other; if one shifts to the blue energy, the second shifts to the red energy. This can be explained by the fact that if the sulfur atom is pulled by a physical force originating from the silver particles (Figure 3b), the C-S-C bond will be compressed and consequently the C-C bond will be stretched, and the reverse is true. As the skeletal modes are not symmetric, this can explain the oscillation behavior as a function of the position. The latter confirms that the two modes depend on the polarization of the local electric field where its gradient changes when the molecules move far from the hotspots and not the magnitude.

**Figure 4:**
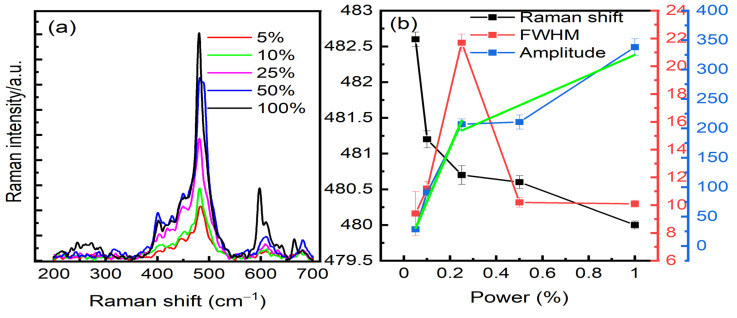
Power-dependent SERS study. (**a**) Power percentage varies between 5% and 100%. (**b**) Parameters (FWHM, Raman shift, amplitude) extracted from the fitting of the band located at 480 cm^−1^ with a Lorentzian function. Linear functions (two green lines) used to fit the variation in the amplitude.

Figure 4a shows the power-dependent SERS study. We varied the power density between 5% and 100%. By fitting the peak located at 480 cm^−1^ that corresponds to the C-N symmetrical stretching, we can extract the amplitude, the FWHM, and the Raman shift, see Figure 4b and Figure S2. The intensity increases linearly faster when we vary the power between 5% and 25% than between 25% and 100%. The variation between 5% and 25% can be fitted with a linear function with a slope larger than the intervals 25–100%. For the Raman shift, a red shift is observed with the increase in power, it shifts very fast between 5% and 25% and then it shifts slowly. The FWHM increases linearly faster between 5% and 25% and then it drops down. Increasing the power could affect the polarizability of the molecule that depends strongly on the orientation of the molecule, especially, when the laser power becomes higher. The latter confirms what we observed in Figure 2d where the Raman shift of C-N stretching bond becomes less sensitive to the weak local electrical field magnitude generated at some positions.

### 3.3. Simulation 

In order to understand the effect of the gap size on the optical enhancement generated by Ag nanoparticles, we performed theoretical studies using COMSOL software. 

COMSOL Multiphysics software is used to execute expanded simulations and to investigate, in particular, the wave–optic interface covering the modeling of electromagnetic fields and waves in the frequency domain. We proceed with the interface by first formulating Maxwell’s equation (Equation (1)):

(1) $$\Delta \times \mu_{r}^{-1}\left(\nabla \times \vec{E}\right)-k_{0}^{2}\left(\varepsilon -\frac{j\sigma }{\omega \varepsilon_{0}}\right)\vec{E}=0$$

where ω, $\mu_{r}$, ε, and σ are the excitation frequency, the relative permeability (fixed to 1), the relative permittivity, and the electrical conductivity, respectively. $\varepsilon_{0}$ and $k_{0}$ represent, respectively, the permittivity and the wave number in free space, being $k_{0}=\omega /c_{0}$ (with $c_{0}$ the speed of light in a vacuum). In these simulations, we consider the relative dielectric permittivities that correspond to the optical frequencies (refractive index model with *n* and *k* real and imaginary refractive indexes, respectively, for the electric displacement), ε = (*n* − i*k*)^2^ (in Equation (1)) [28,29]. Finally, we suppose a perfect matched layer that absorbs the propagation of the wave within the computational region and considering reflections in the interior interface. We use the finite element method to solve and discretize the equation in numerically stable edge elements.

The simulation is performed with the same experimental conditions for the sake of precise prediction of the electromagnetic field distribution in the two-dimensional (2D) model of Ag spherical nanoparticles on TiO_2_. 

The mechanism of electromagnetic field enhancement can be resumed in two steps as follows. The electromagnetic enhancement can be generated first in the vicinity of the Ag nanoparticles where the polaritons are formed. The local field is further amplified, and a dipole is produced conducting to the enhancement of the Raman scattering in the nanogap and a formation of plasmons [30,31]. After, an interactive excitation from the system of the Ag nanoparticles at a resonant frequency (plasmon resonance) generates an enhanced apparent Raman polarizability. Consequently, the calculated enhanced Raman scattered light *G* from the structure of Ag nanoparticles (Equation (2)) is presented as follows:

(2) $$G=\left(\frac{I_{SERS}}{I_{Raman}}\right)\times \left(\frac{N_{Raman}}{N_{SERS}}\right)$$

where $I_{SERS}$ is the intensity of SERS generated by Ag nanoparticle, $I_{Raman}$ is the intensity of Raman in the far-field generated from silicon nanorod, $N_{Raman}$ is the number of molecules in a laser spot generated in the far-field, and $N_{SERS}$ is the number of molecules generated by the SERS signal. $N_{SERS}$ is estimated by taking into account the surface area of Ag nanoparticles.

**Figure 5:**
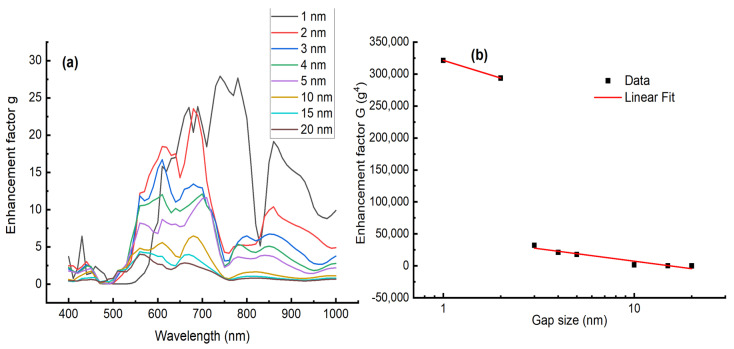
(**a**) Variation in the enhancement factor g as function of the wavelength for different gap size. (**b**) Variation in the enhancement factor g^4^ as function of the gap size between the silver nanoparticles. The red line represents a linear fit to the data. The slope is −91,934 for the gap size range [1–2 nm] and −39,013 for [3–20 nm]. Adj. R-square 0.89518.

Figure 5a shows the variation in the enhancement factor g versus the wavelength for different gap distances of Ag nanoparticles. The enhancement factor g increases by decreasing the gap distance and its maxima also varies in position. Figure 5b shows the variation in the enhancement factor as a function of the gap size for the maximum of enhancement located at 633 nm. The enhancement decreases linearly with the gap size with a slope much larger in the range [1–2 nm] than [3–20 nm]. The observed behavior is in good accordance with the linear decreasing in Raman intensity, as shown in Figure 2c.

**Figure 6:**
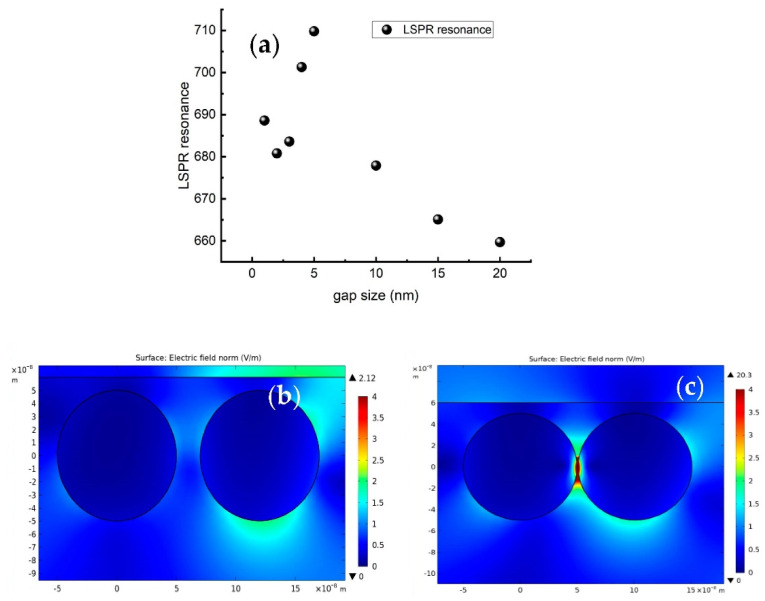
(**a**) LSPR resonance as function of gap size. (**b**,**c**) Mapping of local electric field distribution for a gap size of 1 nm and 20 nm, respectively.

Figure 6a presents the variation in LSPR versus the gap size. We observe a red shift with a fast slope by varying the gap between 2 and 5 nm, then a blue shift with slower slope between 10 and 20 nm. For a LSPR wavelength located at 633 nm that corresponds to the excitation wavelength used in our experimental conditions, we presented a mapping of the optical enhancement for two different gap sizes, 1 nm and 20 nm (see Figure 6b,c). The mapping is performed on 20 × 20 nm of two silver nanoparticles with a diameter 10 nm deposited onto TiO_2_. The enhancement factor g is intensively localized within the gap size of 2 nm; however, it is negligible within the gap size of 20 nm.

## 4. Conclusions

We successfully studied the EM enhancement properties of the silver/TiO_2_ nanostructures by probing SERS spectra. SERS measurement confirmed that EM enhancement is the origin of SERS. The SERS intensity and shift revealed how the vibrational modes in the molecule are affected when the local electric field magnitude and polarization change. A power-dependent SERS study confirmed the change into the polarizability and the irreversibility of the optical enhancement. A theoretical study using finite element method confirmed that the linearity of the gap size is dependent on optical enhancement. A good accordance between the slope transition from the small gap size range to larger gap size between the experiment and the simulation is confirmed.

## Data Availability

Not applicable.

## Supplementary Materials

The following supporting information can be downloaded at: https://www.mdpi.com/article/10.3390/mi13101595/s1, Figure S1: Mapping of the Raman intensity of the two peaks located at 480 and 1623 cm^−^^1^; Figure S2: Fitting of the Raman line located at 480 cm^−1^ excited at different power density.

## References

[1] Park C.G., Kim J.Y., Lee E., Choi H.K., Park W.H., Kim J., Kim Z.H. (2012). Tip-Enhanced Raman Scattering with a Nanoparticle-Functionalized Probe. Bull. Korean Chem. Soc..

[2] Meng L., Huang T., Wang X., Chen S., Yang Z., Ren B. (2015). Gold-coated AFM tips for tip-enhanced Raman spectroscopy: Theoretical calculation and experimental demonstration. Opt. Express.

[3] Dab C., Awada C., Merlen A., Ruediger A. (2017). Near-field chemical mapping of gold nanostructures using a functionalized scanning probe. Phys. Chem. Chem. Phys..

[4] Nelayah J., Kociak M., Stephan O., Geuquet N., Henrard L., de Abajo F.J.G., Pastoriza-Santos I., Liz-Marzan L.M., Colliex C. (2010). Two-Dimensional Quasistatic Stationary Short Range Surface Plasmons in Flat Nanoprisms. Nano Lett..

[5] Awada C., Popescu T., Douillard L., Charra F., Perron A., Yockell-Lelievre H., Baudrion A.L., Adam P.M., Bachelot R. (2012). Selective Excitation of Plasmon Resonances of Single Au Triangles by Polarization-Dependent Light Excitation. J. Phys. Chem. C.

[6] Luceño-Sánchez J.A., Díez-Pascual A.M., Peña Capilla R. (2019). Materials for Photovoltaics: State of Art and Recent Developments. Int. J. Mol. Sci..

[7] Wang K., Yoshiiri K., Rosa L., Wei Z., Juodkazis S., Ohtani B., Kowalska E. (2021). TiO_2_/Au/TiO_2_ plasmonic photocatalyst with enhanced photocatalytic activity and stability under visible-light irradiation. Catal. Today.

[8] Echtermeyer T.J., Milana S., Sassi U., Eiden A., Wu M., Lidorikis E., Ferrari A.C. (2016). Surface Plasmon Polariton Graphene Photodetectors. Nano Lett..

[9] Yang L., Chen G., Wang J., Wang T., Li M., Liu J. (2009). Sunlight-induced formation of silver-gold bimetallic nanostructures on DNA template for highly active surface enhanced Raman scattering substrates and application in TNT/tumor marker detection. J. Mater. Chem..

[10] Martirez J.M.P., Bao J.L., Carter E.A. (2021). First-Principles Insights into Plasmon-Induced Catalysis. Annu. Rev. Phys. Chem..

[11] Yoon J.W., Kim J.-H., Jo Y.-M., Lee J.-H. (2022). Heterojunction between bimetallic metal-organic framework and TiO_2_: Band-structure engineering for effective photoelectrochemical water splitting. Nano Res..

[12] Seino M., Henderson E.J., Puzzo D.P., Kadota N., Ozin G.A. (2011). Germanium nanocrystal doped inverse crystalline silicon opal. J. Mater. Chem..

[13] Gogoi D., Namdeo A., Golder A.K., Peela N.R. (2020). Ag-doped TiO_2_ photocatalysts with effective charge transfer for highly efficient hydrogen production through water splitting. Int. J. Hydrogen Energy.

[14] Huynh W.U., Dittmer J.J., Alivisatos A.P. (2002). Hybrid nanorod-polymer solar cells. Science.

[15] Yang L., Sang Q., Du J., Yang M., Li X., Shen Y., Han X., Jiang X., Zhao B. (2018). A Ag synchronously deposited and doped TiO_2_ hybrid as an ultrasensitive SERS substrate: A multifunctional platform for SERS detection and photocatalytic degradation. Phys. Chem. Chem. Phys..

[16] Zhao X., Zhang W., Peng C., Liang Y., Wang W. (2017). Sensitive surface-enhanced Raman scattering of TiO_2_/Ag nanowires induced by photogenerated charge transfer. J. Colloid Interface Sci..

[17] Mills A., Le Hunte S. (1997). An overview of semiconductor photocatalysis. J. Photochem. Photobiol. A Chem..

[18] Kolwas K., Derkachova A. (2020). Impact of the interband transitions in gold and silver on the dynamics of propagating and localized surface plasmons. Nanomaterials.

[19] Awada C., Hajlaoui T., Al Suliman N., Dab C. (2022). Heterogeneous Nanoplasmonic Amplifiers for Photocatalysis’s Application: A Theoretical Study. Catalysts.

[20] Zhou L., Zhou J., Lai W., Yang X., Meng J., Su L., Gu C., Jiang T., Pun E.Y.B., Shao L. (2020). Irreversible accumulated SERS behavior of the molecule-linked silver and silver-doped titanium dioxide hybrid system. Nat. Commun..

[21] Yang L., Jiang X., Ruan W., Yang J., Zhao B., Xu W., Lombardi J.R. (2009). Charge-Transfer-Induced Surface-Enhanced Raman Scattering on Ag−TiO_2_ Nanocomposites. J. Phys. Chem. C.

[22] Jaramillo-Fierro X., Capa L.F., Medina F., González S. (2021). DFT Study of Methylene Blue Adsorption on ZnTiO3 and TiO_2_ Surfaces (101). Molecules.

[23] Zhao Z., Liu G., Li B., Guo L., Fei C., Wang Y., Lv L., Liu X., Tian J., Cao G. (2015). Dye-sensitized solar cells based on hierarchically structured porous TiO_2_ filled with nanoparticles. J. Mater. Chem. A.

[24] Shafi M., Zhou M., Duan P., Liu W., Zhang W., Zha Z., Gao J., Wali S., Jiang S., Man B. (2022). Highly sensitive and recyclable surface-enhanced Raman scattering (SERS) substrates based on photocatalytic activity of ZnSe nanowires. Sensors Actuators B Chem..

[25] Yang J., Zhou L., Wang X.-Y., Song G., You L.-J., Li J.-M. (2020). Core-satellite Ag/TiO_2_/Ag composite nanospheres for multiple SERS applications in solution by a portable Raman spectrometer. Colloids Surfaces A Physicochem. Eng. Asp..

[26] Shim K.-D., Jang E.-S. (2018). SERS Signal Enhancement of Methylene Blue-embedded Agglomerated Gold Nanorod@SiO_2_ Core@Shell Composites. Bull. Korean Chem. Soc..

[27] Vu X.H., Dien N.D., Ha Pham T.T., Trang T.T., Ca N.X., Tho P.T., Vinh N.D., Van Do P. (2020). The sensitive detection of methylene blue using silver nanodecahedra prepared through a photochemical route. RSC Adv..

[28] Johnson P.B., Christy R.W. (1972). Optical constants of the noble metals. Phys. Rev. B.

[29] Ghosh G. (1999). Dispersion-equation coefficients for the refractive index and birefringence of calcite and quartz crystals. Opt. Commun..

[30] Awada C., Dab C., Grimaldi M.G., Alshoaibi A., Ruffino F. (2021). High optical enhancement in Au/Ag alloys and porous Au using Surface-Enhanced Raman spectroscopy technique. Sci. Rep..

[31] Dab C., Thomas R., Ruediger A. (2018). Modeling of the surface plasmon resonance tunability of silver/gold core-shell nanostructures. RSC Adv..

