# Supplementary material for: Plasmonic Enhanced SERS in Ag/TiO2 Nanostructured Film: An Experimental and Theoretical Study
Source: Micromachines (Basel). 2022 Sep 25;13(10):1595. doi: 10.3390/mi13101595 (PMC9610157; doi:10.3390/mi13101595)
Supplement: Supplementary file 1 [file micromachines-13-01595-s001.zip › micromachines-1898867-supplementary.pdf]

Supplementary Materials

Plasmonic enhanced SERS in Ag/TiO_2 nanostructured film: an experimental and theoretical study

Chawki Awada

Department of Physics, College of Science, King Faisal University, P.O. Box: 400, Al-Ahsa, 31982, Saudi Arabia

Figure S1: Mapping of the Raman intensity of the two peaks located at 480 and 1623 cm^{-1}.

- Mapping of 480 cm^{-1}

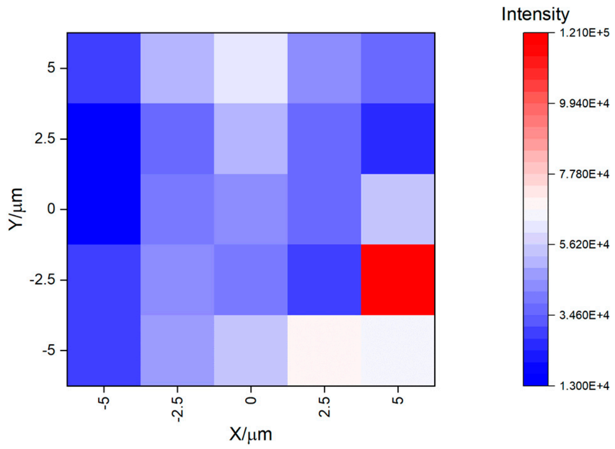

- Mapping at 1623 cm^{-1}

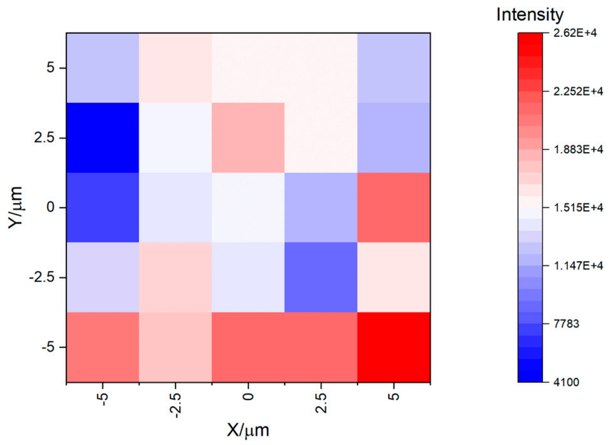

Figure S2: Fitting of Raman line located at 480 cm^{-1} excited at different power density.

- 5%

Peak Analysis

Data Set:[A20Ag40powers]"20-Ag-40_power_sers _100%"!W"Subtracted_Data Y1"
BaseLine:Constant Date:10/3/2021
Chi^2=1.55857E+01 Adj. R-Square=9.77751E-01 # of Data Points=633
SS=9.69429E+03 Degrees of Freedom=622

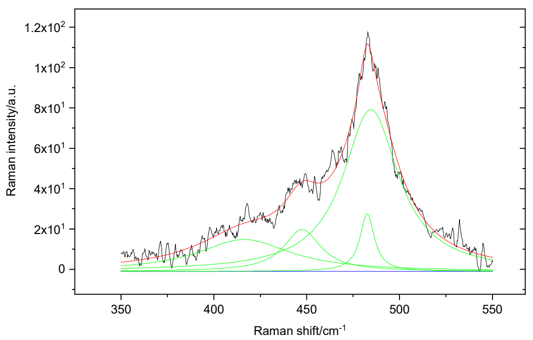

Fitting Results

Peak Index	Peak Type	Area Intg	Area IntgP	Center Grvty	Max Height	FWHM
1	Lorentz	1195.31028	18.921	416.34346	15.77293	60.91875
2	Lorentz	726.7771	11.50442	447.53165	20.75584	24.13536
3	Lorentz	406.37705	6.43269	482.576	28.45746	9.40496
4	Lorentz	3988.90819	63.14189	484.53814	80.16414	36.35683

- 10%

Peak Analysis

Data Set:[A20Ag40powers]"20-Ag-40_power_sers _100%"!AA"Subtracted_Data Y1" Date:10/3/2021
BaseLine:Constant
Chi^2=1.86573E+01 Adj. R-Square=9.82273E-01 # of Data Points=780
SS=1.43288E+04 Degrees of Freedom=768

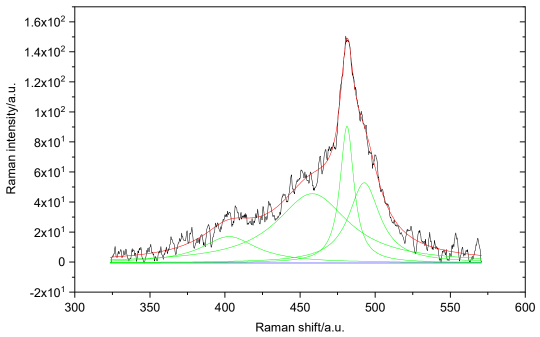

Fitting Results

Peak Index	Peak Type	Area Intg	Area IntgP	Center Grvty	Max Height	FWHM
1	Lorentz	1061.25551	13.01562	402.70564	17.47854	44.36848
2	Lorentz	3599.0743	44.14035	458.15342	46.03063	58.48692
3	Lorentz	1548.65939	18.99332	481.24866	90.91111	11.1934
4	Lorentz	1944.71601	23.8507	492.789	53.30452	25.0892

- 25%

Peak Analysis

Data Set:[A20Ag40powers]"20-Ag-40_power_sers _100%"!AE"Subtracted_Data1 Y3" Date:10/3/2021
BaseLine:Constant
Chi^2=4.45936E+01 Adj. R-Square=9.87099E-01 # of Data Points=734
SS=3.21966E+04 Degrees of Freedom=722

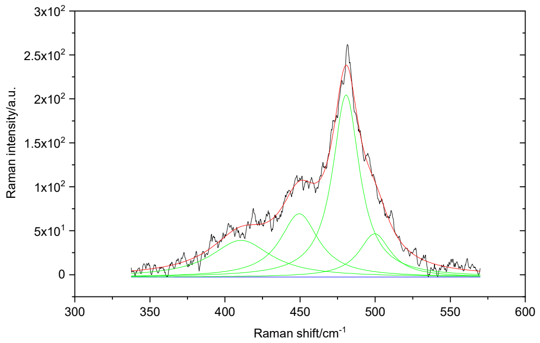

Fitting Results

Peak Index	Peak Type	Area Intg	Area IntgP	Center Grvty	Max Height	FWHM
1	Lorentz	2717.31807	18.91238	410.70763	41.79399	48.73791
2	Lorentz	3216.46584	22.38642	449.48806	72.06298	31.04335
3	Lorentz	6618.43591	46.06394	480.67809	207.1306	21.70025
4	Lorentz	1815.71312	12.63726	499.77443	49.32062	25.53921

- 50%

Peak Analysis

Data Set:[A20Ag40powers]"20-Ag-40_power_sers _100%"!AI"Subtracted_Data1 Y4" Date:10/4/2021
BaseLine:Constant
Chi^2=4.71902E+01 Adj. R-Square=9.94443E-01 # of Data Points=714
SS=3.29859E+04 Degrees of Freedom=699

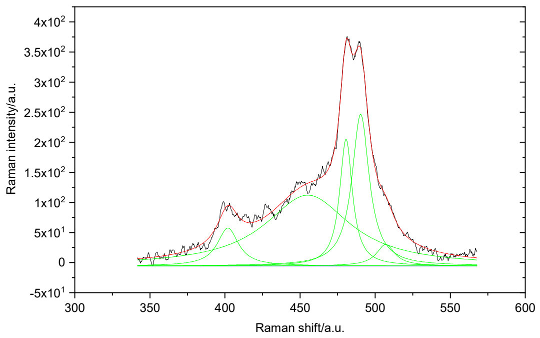

Fitting Results

Peak Index	Peak Type	Area Intg	Area IntgP	Center Grvty	Max Height	FWHM
1	Lorentz	1579.32701	7.44129	401.94281	63.06984	16.97555
2	Lorentz	10321.86936	48.63338	455.1229	117.67485	68.78086
3	Lorentz	3277.71224	15.44354	480.61633	210.78952	10.20885
4	Lorentz	5259.31851	24.78024	490.32683	251.92124	13.89304
5	Lorentz	785.61081	3.70155	507.01737	35.81264	14.74129

- 100%

Peak Analysis

Data Set:[A20Ag40powers]"20-Ag-40_power_sers _100%"!AM"Subtracted_Data" Date:10/[illegible]/2021

BaseLine:Constant

Chi^2=6.78860E+01 Adj. R-Square=9.92990E-01 # of Data Points=643

SS=4.28360E+04 Degrees of Freedom=631

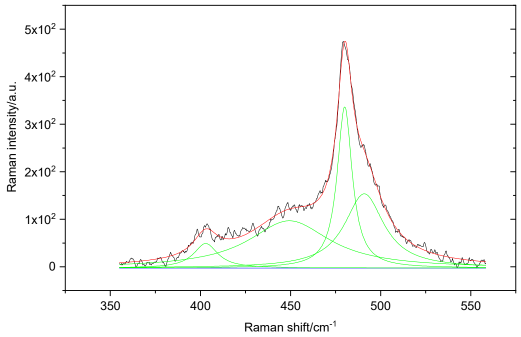

Fitting Results

Peak Index	Peak Type	Area Intg	Area IntgP	Center Grvty	Max Height	FWHM
1	Lorentz	1224.45579	6.29193	402.94973	51.79138	16.18129
2	Lorentz	7124.78256	36.61107	449.34427	99.46514	54.84704
3	Lorentz	5209.03045	26.76688	480.04365	339.0889	10.11773
4	Lorentz	5902.4625	30.33012	490.90891	156.28895	26.49933
